# Statistics of Mortality in Different Countries

**Published:** 1856-01

**Authors:** 


*Statistics of Mortality in Different Countries.*—According to the reports from European countries, and the United States' census returns for 1850, the ratio of mortality in the several countries may be stated as follows:

| | | | | |
|---|---|---|---|---|
| England, | deaths, | 2.2 | per cent. | per annum. |
| France, | " | 2.4 | " | " |
| Prussia, | " | 2.7 | " | " |
| Austria, | " | 3.1 | " | " |
| Russia, | " | 3.5 | " | " |
| U. States, | " | 1.4 | " | " |

The foregoing figures, which make the mortality of the United States compare so favorably with that of the different countries of Europe, are taken from a well written article in the New York *Herald.* The ratio for this country is deduced from the census of 1850, and by no means corresponds with the facts developed in the following extract of an address by Dr. Hubbard, of Connecticut, which we copy from the Buffalo Medical Journal, viz:

"The Registration law, now in operation in this State, is in the main excellent; and, if universally complied with, would develop a mass of valuable facts which could not fail of exerting a great and lasting influence on our prosperity.

"It is intended to accomplish two great objects:

"First. *To preserve the name, and afford the means of identifying the connections, and some facts concerning the personal history of every person who is born, marries, or dies in the community.*

"Second. *To determine how health, life, and longevity are affected by age, sex, condition, and occupation; by climate, season, and place of residence; and by the diseases to which, under any circumstances, man may be subject.*

"To accomplish the first object, certificates of birth should, in all cases, state the *maiden name of the mother*, the *national-*

*ity of both parents*, and, as children are not often named when the return is made, it should state the *number* of the child, whether first, or second, &c., in addition to those items now required.

"Marriage certificates should also state, in addition, the *names and residences* of *the parents of both parties*, and the names of witnesses.

"Certificates of death should also state, in addition, the *names of the parents of the deceased*, and their *nationality*. In order to determine identity, it is necessary that these, and all the facts now required, should be recorded with exactness. Physicians, in too many cases, omit one or more of these facts, without reflecting that, perhaps, the very one which they consider of so little consequence may be hereafter of the first importance to that individual or his friends, to say nothing of the loss which science sustains in the omission of a fact.

"Records of this kind are of great importance in the various civil relations of society; and will secure to all classes numerous legal rights. It is useful to all persons, and to some it is of the greatest importance, to be able to prove, in a legal way, their age and place of birth; and equally important is the day of death, and the particulars of the marriage contract.

"Who does not know of individuals who have failed to obtain their rights of property, or have suffered in reputation, for the want of such legal proof of events and identity, as this law proposes to furnish?

"A family, once resident in New Haven, the undoubted heirs, by the mother's side, of a princely fortune in England, failed to receive it, for the reason that a *single fact* was wanting to complete the chain of evidence otherwise conclusive; and it is well known that millions of property in England, rightfully belonging to parties in this country, have been forfeited to the British Crown, because no legal record of births, marriages, and deaths had been kept.

"The widow of the late Dr. Dwight, President of Yale College, was for many years unable to procure the pension to which her husband's services in the army of the Revolution entitled her, for the reason that she had no proof of her marriage, no record having been made, and the witnesses being dead; she finally obtained it, however, through the aid of Joel R. Poinsett, while Secretary of War. He ordered it to be granted, on the ground that it was not to be supposed that so wise and so good a man as his old and venerated instructor, would have lived all his life with a woman who was not his wife. How many families in this State would have been made glad, or have been saved from expensive litigation and pecuniary ruin, had such a plan of registration been faithfully carried out.

"The records of New Haven have been repeatedly brought into requisition within two years, as legal evidence in suits affecting the social rights of individuals. Copies have been required in order to settle estates in England, Germany, Cuba, New York, and Massachusetts, besides for other purposes within the State; and I presume similar facts are known in other parts of the State. So far as my observation extends, the law is increasingly popular with the more intelligent portions of every community, who justly regard it as capable of conferring upon the State numerous benefits, the importance of which can not be estimated.

"To accomplish the second object, the record should show a class of facts different from those necessary to prove identity, though, in some particulars, they are the same; but as these are all included in the present law, I need not mention them here, except to remark that the attending physician should, in all cases, be careful to state in a certificate of birth, the *age of the parents*, which many omit to do: this appears at first but a trifling matter, yet in future years such facts will prove of great service in determining the laws of population.

"We shall know by these means what is the proportion of births and deaths among our foreign population, an item at present of peculiar interest; also, of what diseases the foreign born and their children die. Forming, as they do everywhere, a large and increasing element in our population, frequently bringing with them the seeds of disease and death, this becomes a matter of serious import.

"The register in New Haven exhibits the fact, I believe, that a majority of the children who die there are of foreign parentage, and that of these the larger part die between three and five years of age. If this statement is correct, and should be corroborated in other large towns—the principal foci of the foreign population—it would be an interesting and useful fact. It is easy to see that by the accumulation of facts, registration will lead to the adoption of such measures as will aid in the diminution of sickness—(and one-half of all that occurs is believed to be unnecessary and preventable)—in the security of life, in the improvement of the general physical condition of the people, and in promoting their greatest happiness.

"Among the other important considerations connected with this subject is its bearing on Life Insurance.

"It was long ago ascertained in Europe that the reproduction, the life, the sickness and death of man, are regulated by certain fixed and natural laws, which vary, of course, according to the individual, and the circumstances by which he is surrounded. These laws had not yet been investigated in their application to man in the circumstances in which he is placed in this country—neither can it be done in the present state of knowledge on this subject.

"It is evident, however, that many facts exist, which render the operation of these laws peculiar to ourselves; and it is highly desirable, on this account, also, that a system of registration of human life, by which they may be obtained, should be faithfully carried into operation.

"Life insurance, as common in England as insurance on property, is becoming a very important interest in this country; and it has always been regretted that insurance companies are obliged to base all their operations on either the Carlisle or other tables of mortality, prepared in England many years ago, and which never afforded an adequate estimate of the probabilities of life on this continent.

"'Such records would enable us to construct tables of mortality, containing an individual fund of statistical information, showing the various influences in operation among us, which tend to increase or diminish our population, the comparative value of life among males and females, and of persons existing under different circumstances and conditions; the comparative prevalence of health and disease and of death, in the different seasons of the year, in different localities, and in the different periods of life.'

"Until we have such a class of facts, we cannot know the wants of our population, nor tell where to apply remedies in order to ameliorate their condition—to improve the general health of community—promote the security of life, and add to the number of its years. At present, our exertions must be influenced by, and be made upon, comparatively uncertain theory and conjecture; and, of course, may produce erroneous results.

"Registration has developed some interesting facts of a more strictly sanitary character. In England, it has been found that in those rural districts in which under-drainage had been within a few years generally adopted, the number of deaths has much diminished, while the cases of sickness were fewer and shorter; and in the large towns, the difference between the ratio of deaths in the undrained, crowded localities, and the better portions of the cities, was very striking. In the report of Mr. Glover, superintending medical inspector of the London Board of Health, on the common and model lodging-houses of

London (with reference to epidemic cholera in 1854) it is stated that, in 1853, there were registered houses of this kind, accommodating about 30,000 persons, yet, during the year, *only ten cases of fever* occurred. Considering the class of persons inhabiting these houses, it must be acknowledged that three cases of fever in every 10,000 of such persons, is an almost incredible amount of sickness of this character.

"'In all the houses, registered and unregistered, there were, in the first nine months of last year, 72 cases of cholera, and 61 deaths—an amount of sickness, all things considered, astonishingly small.' 'With respect to the health of the inmates of the model lodging-houses, it appears, from the various reports, that these houses have enjoyed all but a complete exemption from the cholera, the mortality among the inmates having been only in the ratio of about 26 in 10,000; whereas the mortality from cholera in the potteries, Kensington, was in the ratio of 259 in every 10,000; and in Bermondsey, 162 in 10,000.'

"In a comparison of the bills of mortality in London with those of Boston, which has always been cited as a model city for cleanliness and sobriety, we find a remarkable coincidence. In London, 32 per cent. of the deaths are those of children under five years of age; the average age of all, at death, is twenty-six and a half years, and the annual rate of mortality for the whole population is 1 in 40.

"'In Boston, from 1840 to 1845, 46.62 per cent. of all the deaths were those of children under five years of age; and in some classes of the population, more than 62 per cent. were under that age; the average age of all that died in the same period was 21.43 years, and of the Catholic burials, 13.43 *years only*. The rate of mortality for the whole population, for the last nine years, was 1 in 39; and for the last year (1849) 1 in 26.' Showing that London, with its two millions of people, supplied with water from the Thames, into which the enormous accumulation of waste and dead animal and vegetable matter—the blood and offal of slaughter-houses—the drainage from die-works, bone-boiling houses—and a thousand nameless pollutions, all find their way—with its crowded streets and grave-yards, its foul cess-pools and hopeless pauperism—is as healthy a city as Boston, and in some respects more so.

"Some of our other cities suffer still more by the comparison. The annual average mortality for the last eight years was:

| | | | |
|---|---|---|---|
| In Philadelphia, . . . | 1 death in | 42 | inhabitants. |
| " Boston, . . . . . . | 1 " | 39 | " |
| " Baltimore, . . . . | 1 " | 36 | " |

In Chicago, . . . . . 1 " 29 inhabitants.
" New York, . . . . 1 " 25 "

" Last year the average ratio of deaths in Chicago was 1 in 18.3 of the population.

" The high ratio in the two latter cities is owing entirely to the larger proportion of immigrants constantly arriving there; while in Chicago full one-half the entire population are foreign born, and there is always present a floating population of several thousands, many of whom are yet suffering from the debilitating effects of a long voyage, and destitute of every comfort and convenience of life.

" Doubtless among the principal reasons for the large mortality in this country, may be mentioned the great and frequent changes of temperature at all seasons; the intense and prolonged heat of summer, favoring rapid decomposition, and causing diseases of the bowels, as diarrhœa, dysentery, cholera, &c.; the equitable and restless state of our population, containing a large proportion of foreigners, among whom affections of the bowels and lungs seem to be particularly fatal. It is to be expected, then, that the deaths in proportion to the population would be more numerous, and the average age at death lower, than in the slow-going population, and more equable, temperate climates of the old world.

" Says Mr. Chadwick: 'The average of the whole of the living population in America, so far as it can be deduced from the census returns, is only 22 *years and* 2 *months*. Notwithstanding the earlier marriages and the extent of emigration, and the general increase of population, the whole circumstances appear to me to prove this to be the case of a population, depressed to this low age, chiefly by the greater proportionate pressure of the causes of disease and premature mortality. The proportionate numbers at each interval of age in every 10,000 of the population of the United States, England and Wales, are as follows:

| | United States. | England and Wales. |
|---|---|---|
| Under 5 years, | 1,744 | 1,324 |
| 5 and under 10, | 1,417 | 1,197 |
| 10 " 15, | 1,210 | 1,089 |
| 15 " 20, | 1,091 | 997 |
| 20 " 30, | 1,816 | 1,780 |
| 30 " 40, | 1,160 | 1,289 |
| 40 " 50, | 732 | 959 |
| 50 " 60, | 436 | 645 |
| 60 " 70, | 245 | 440 |
| 70 " 80, | 113 | 216 |

| | | |
|---|---|---|
| 80 and under 90, | 22 | 59 |
| 100 and upward, | 4 | 5 |

Average age of all living, 22 yrs. and 2 mos. 26 yrs., 7 mos.

"'It may be observed,' he adds, 'that while in England there are 5,025 persons between 15 and 50, who have 3,610 children, or persons under 15; in America there are 4,789 persons living, between 15 and 50 years of age, who have 4,371 children dependent upon them.

"'In England there are, in every 10,000 persons, 1,365 who have obtained over 50 years' experience; in America, there are only 830.

"'The moral consequences of the predominance of the young and passionate in the American community, are attested by observers to be such as have already been described in the General Sanitary Report, as characteristic of those crowded, filthy and badly administered districts of England, where the average duration of life is short, the proportion of the very young great, and the adult generation transient. The adult population of America, it has been shown, is younger than in England, and if the causes of early death were to remain the same, it may be confidently predicted that the American population would remain young for centuries.'"

